# Small-Angle X-ray Scattering (SAXS) Used for the Identification of Nicomorphine Polymorphic Changes at the Early Stage to Avoid Varied Stability and Possible Side Effects

**DOI:** 10.3390/ph17030375

**Published:** 2024-03-15

**Authors:** Nermina Malanovic, Giovanni Birarda, Simone Eder, Heidrun Gruber-Woelfler, Franz Reiter, Krunoslav Juraic, Aden Hodzic

**Affiliations:** 1 Institute of Molecular Biosciences, University of Graz, 8010 Graz, Austria; 2 Field of Excellence BioHealth, University of Graz, 8010 Graz, Austria; 3 Bio TechMed Graz, 8010 Graz, Austria; 4 Elettra Synchrotron, Basovizza, 34149 Trieste, Italy; giovanni.birarda@elettra.eu; 5 Institute of Pharmaceutical Sciences, University of Graz, 8010 Graz, Austria; simone.eder@rcpe.at; 6 Research Center Pharmaceutical Engineering GmbH, 8010 Graz, Austria; woelfler@tugraz.at; 7 Institute of Process and Particle Engineering, Graz University of Technology, 8010 Graz, Austria; 8 G.L. Pharma GmbH, 8502 Lannach, Austria; franz.reiter@gl-pharma.at; 9 Rudjer Boskovic Institute, 10000 Zagreb, Croatia; kjuraic@irb.hr; 10 Central European Research Infrastructure Consortium (CERIC-ERIC), Basovizza, 34149 Trieste, Italy

**Keywords:** polymorphism, crystallinity, purity, solid-state drugs, powder, small-angle X-ray scattering, Raman, FTIR, dissolution, DSC

## Abstract

In this paper, we present the identification of polymorphisms at an early stage, identified by applying non-standard methods such as SAXS. We provide an analytical approach to polymorphism in the quality/purity of an active pharmaceutical ingredient (API), supplied to a generic company by two different suppliers (i.e., manufacturers). Changes in thermodynamic polymorphism firstly become visible in traces in the larger crystal lattices, which are visible on the SAXS spectrum only using the logarithmic scale, as shown in the result figures. Hence, we are here on the trail of the beginning of a new polymorph in nicomorphine, whose crystal waviness at the early stage is visible only in the additional symmetrical peaks identified and calculated using SAXS, while the chemical analyses excluded all kinds of chemical impurities. The chemical and structural properties were studied using the following techniques: SAXS, WAXS, DSC, dissolution, Raman spectroscopy, and FTIR. Only the SAXS technique could identify crucial differences and calculate the additional signals related to giant crystals, whilst a standard method such as WAXS showed none, and nor did the chemical analyses, such as Raman spectroscopy and FT-IR. This means that due to water in crystallization (known in nicomorphine) or thermodynamic waviness, the formation of the new polymorph starts first in traces, which become visible at larger distances from the crystal lattice, detectible only in the SAXS range. This is a very important premise and hypothesis for further research, and we believe that this work lays a new stone in understanding the origin of new unknown polymorphs and their mixtures. Therefore, the aim of this work is to show that the use of non-standard methods (i.e., SAXS) can be of great benefit to API analysis and the identification of polymorphic changes in the early phase, which can cause varied stability, solubility and bioavailability and thus different therapeutic effects or side effects.

## 1. Introduction

The quality of medicines, particularly their crystalline purity in the solid state or polymorphism, is very important to pharmaceutical products and their actual efficacy [1]. To test and detect this in a timely manner, various well-known analytical methods are used. In order to detect these phenomena at an early stage, it is good to use techniques with a different resolution lengthscale of analysis, such as SAXS in this case. In this work, we managed to detect the beginning of the polymorphic transformation of nicomorphine at a very early stage, which is visible only on the logarithmic scale of SAXS but not using other methods.

The fact is that nowadays, most pharmaceutical companies deal with different suppliers of active pharmaceutical ingredients (APIs). Prices and purity play an important role, especially when choosing a supplier. The API quality and crystalline purity must be satisfied, otherwise the final product not only causes manufacturing problems (e.g., tableting or dissolution, stability) [1,2,3] but can also induce possible therapeutic side effects. It is generally known that several processes (e.g., high-energy milling) can alter the crystalline state of an unstable API. However, APIs with larger or smaller molecules may contain more than one crystalline form [4]. Specific crystalline polymorphic forms are associated with a small-molecule therapeutic effect [5] or with a protein [6,7]. The meaning of the word “polymorphism” is of Greek origin, where polus = many and morph = shape. Different polymorphs have not only different solubilities and dissolution rates but also different stability and bioavailability, which can directly influence different side effects or therapeutic effectiveness [8]. The physical properties that differ for each crystal form are the packing, thermodynamics and spectroscopic effects of the API polymorphism on medication bioavailability, which are, within manufacturing, considered in the regulatory framework of new drug delivery systems [9,10,11] and known products like generics [9]. 

X-ray techniques are very suitable for providing information about API polymorphism [12,13]. In addition, to obtain proper quality control, more than one technique is required [14,15,16]. Understanding the relationship between the properties of pharmaceutical solids and their physical structures is therefore important in choosing the most appropriate form of an API for drug product development [17,18]. Thus, we propose a multi-technical approach based on chemical information, dissolution and crystal structure. Small-angle X-ray scattering (SAXS) is presented in this work to be highly useful for API structural analytics based on its identification of the fingerprint [19,20,21] of the crystal structure seen only in the small-angle range. The data are obtained simultaneously using two separate detectors, which collect the X-ray signal from the analyzed sample at two different angles, the small angle (SAXS) and the wide angle (WAXS). Interestingly, the standard-method WAXS does not detect differences, but SAXS analysis shows changes visible at the intensity of the logarithmic scale. SAXS is not a common technique used for crystalline purity detection, but the aim of this work is to show the usefulness of non-standard methods with an extended resolution scale. In addition, the SAXS data match differential scanning calorimetry (DSC) data since both techniques are physically structural-based, compared with all other chemical analytics, which do not serve API differences. Hence, WAXS, Raman spectroscopy and FT-IR, as standard analytical techniques, did not display any significant differences between the two APIs provided by the two producers.

## 2. Results and Discussion

### 2.1. SWAXS

**Figure 1:**
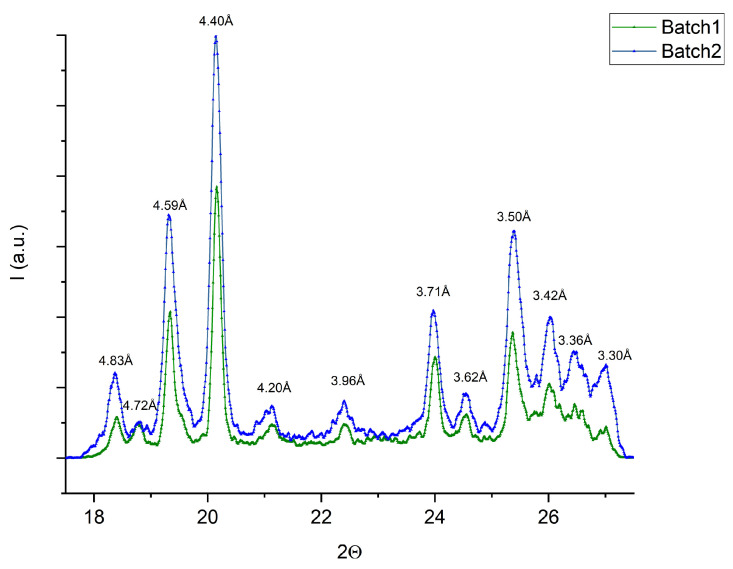
WAXS data, displaying signals for the two APIs.

To observe differences between the two batches of nicomorphine, SWAXS was performed first, since it is particularly sensitive to impurities. At first, mainly identical Bragg peaks were detected in both batches in the WAXS angular range, displaying the same q-values that are the fingerprint of the materials (Figure 1). The APIs displayed visible Bragg peaks in the angular WAXS range of 17° < 2θ < 27°. The WAXS fingerprints appear according to the structural crystalline forms. Such instrumentation cannot detect any scattering signal beyond the diffraction angle 2θ = 27°. The APIs of both batches in the WAXS spectrum serve crystallinity peaks at 4.8, 4.7, 4.5, 4.4, 4.2, 4.0, 3.7, 3.6, 3.5, 3.4 and 3.3 Å. All calculated peak values (see Figure 1) for both APIs are comparable, remained in the same positions in this angular range, and indicate a higher degree of structural order (crystallinity), not an amorphous state. Such a stronger crystallinity could yield a higher stability of the APIs. 

**Figure 2:**
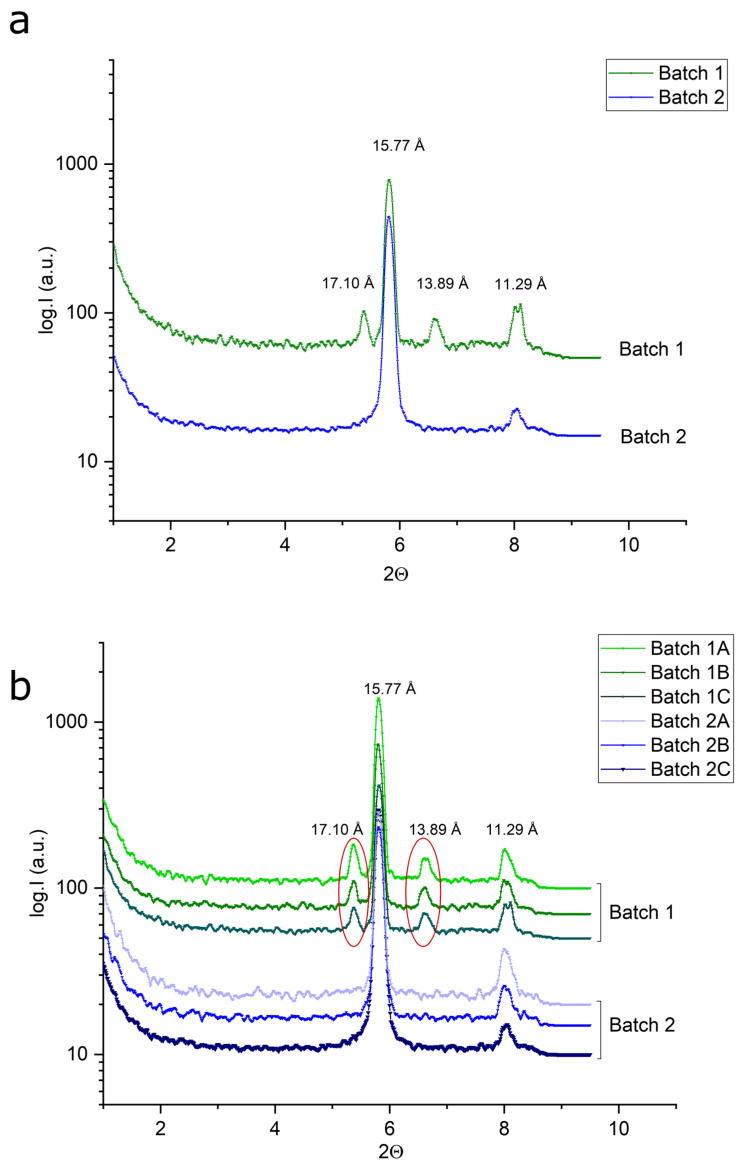
SAXS data with different signals for the two API batches: (**a**) single representative trace, (**b**) triplet measurements. The additional different signals are highlighted by red circles.

Thus, according to the WAXS data, one can assume that the APIs from different producers were in crystalline form without additional impurities. However, the additional polymorphic impurity found in the SAXS region (see Figure 2) did not influence the API structure in the WAXS region, since all crystalline peaks showed the same values (Figure 1) in the WAXS spectrum.

**Figure 3:**
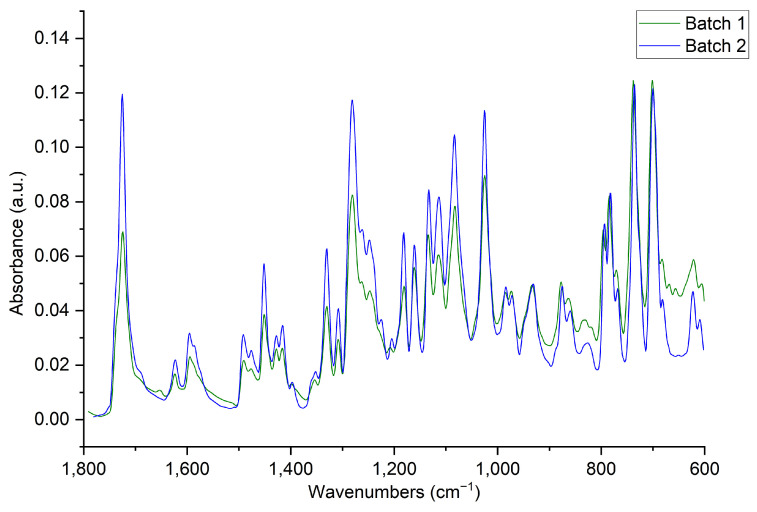
Representative FT-IR spectra displaying almost identical signals for the two API batches.

Furthermore, the SAXS region of our analytics displays an additional material internal order [8]. Figure 2 shows spectra with a 2*θ*-axis (x-scale) in relation to the 2*θ* scattering angle via the equation q = 4π sin θ/λ versus the scattering intensity (y-axis). The reason for the small-angle X-ray signal of scattering [22] is the electron density difference in a material, such as that which occurs at interfaces (i.e., in a powder between air and solid). At the same time, large crystal lattice signals can also appear in the SAXS region in the form of Bragg peaks. For that reason, our samples (Figure 2) show Bragg peaks in the SAXS scale that correspond to the crystalline structure of the API. The data from batches 1 and 2 are displayed in a logarithmic y scale (Figure 2 and Figure 3) to better visualize the Bragg peaks in the SAXS region.

The SAXS signal of the APIs from batch 1 (producer 1) display peaks by 17.1 and 13.9 Å, while batch 2 (producer 2) does not display such differences in order or polymorphic impurities (Figure 2). The SWAXS measurements were repeated and reproduced with triplet measurements (Figure 2b).

In general, the advantage of SAXS and WAXS methods is reflected in the ability of X-rays to pass through the solid material and provide information about the internal and external morphology at once. This is not the case with all spectroscopic methods, which can mainly only analyze solids at the surface (such as FTIR and Raman). However, the disadvantage of SAXS and WAXS is that they are only physical structure methods, without the possibility of direct analysis of chemical groups, except with peak references. An additional disadvantage of both SAXS and WAXS is their limitation in the scale of detection resolution, as SAXS is only relevant to small scattering angles and WAXS only to wide scattering angles (the scale at which fingerprints of organic molecules should be present). For this reason, the combination of SAXS and WAXS into SWAXS is a very powerful tool where at the WAXS scale we can see the fingerprints of materials and at the SAXS scale we can also see the self-assembly structure or fingerprints of larger structural order as here in our work.

### 2.2. ATR-FTIR

Next, we performed ATR-FTIR on the two batches. The analysis revealed no variation between the IR spectra from multiple measurements, within one batch or between the batches. The representative spectrum for both batches is shown in Figure 3. The spectra show bands between 1360 and 1310 cm^−1^, which are attributed to C-N stretching vibrations arising from the aromatic tertiary amine bond in pyridine. In addition, the band between 1190 and 1130 belongs to a secondary amine, i.e., to non-aromatic but aliphatic and heterocyclic C-N stretching. The bands between 1500 and 1600 cm^−1^ belong to an aromatic ring. Methyl C-H_3_ vibrations are between 1400 and 1450 cm^−1^ and belong to the 1st methyl group. The C-H vibrations from aromatic rings are visible between 1200 and 1400 cm^−1^. Carbon–oxygen single bonds are visible between 1200 and 1000 cm^−1^. The pyridine ring can be identified at 700 cm^−1^. The stretching of cyclic ethers, C-O-C, can be recognized between 1140 and 1070 cm^−1^. In addition, the epoxy C-O-ring stretching shows bands between 890 and 800 cm^−1^. The spectra indicate that no chemical difference between the two API batches is visible according to FTIR analytics.

### 2.3. Raman

**Figure 4:**
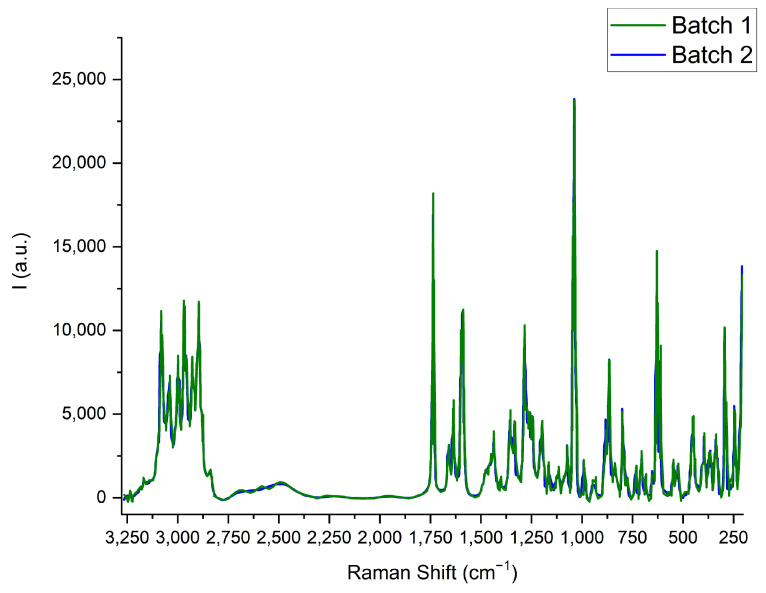
Representative Raman spectra displaying equal signals for the two API batches.

Furthermore, Raman spectra were recorded with the two batches. In fact, the spectral signature in the calculated Raman spectra also does not show significant differences between both batches of nicomorphine (Figure 4). 

The spectra show several bands with small bandwidth, characteristic of crystalline conformations. A tight attribution of all nicomorphine normal modes is in concordance with the aim of the present work, since no chemical difference appears. However, a simple comparison with the Raman spectra of morphine and picolinic acid [23,24] found in the literature can be performed. According to this literature, the Raman bands between 3250 and 2750 cm^−1^ can be attributed to C-H stretching. In the region of 2750 to 2240 cm^−1^, overtone vibrations of features are likely recorded. The signal at 1750 cm^−1^ instead can attributed to the carboxyl C=O symmetric stretching from picolinic acid. The high intensity at about 1000 cm^−1^ can be attributed to picolinic acid ring breath C- stretching modes. Finally, the features at about 600 cm^−1^ allow for a combination of C-C and C-N bending and stretching modes (see Figure 4).

### 2.4. Dissolution

**Figure 5:**
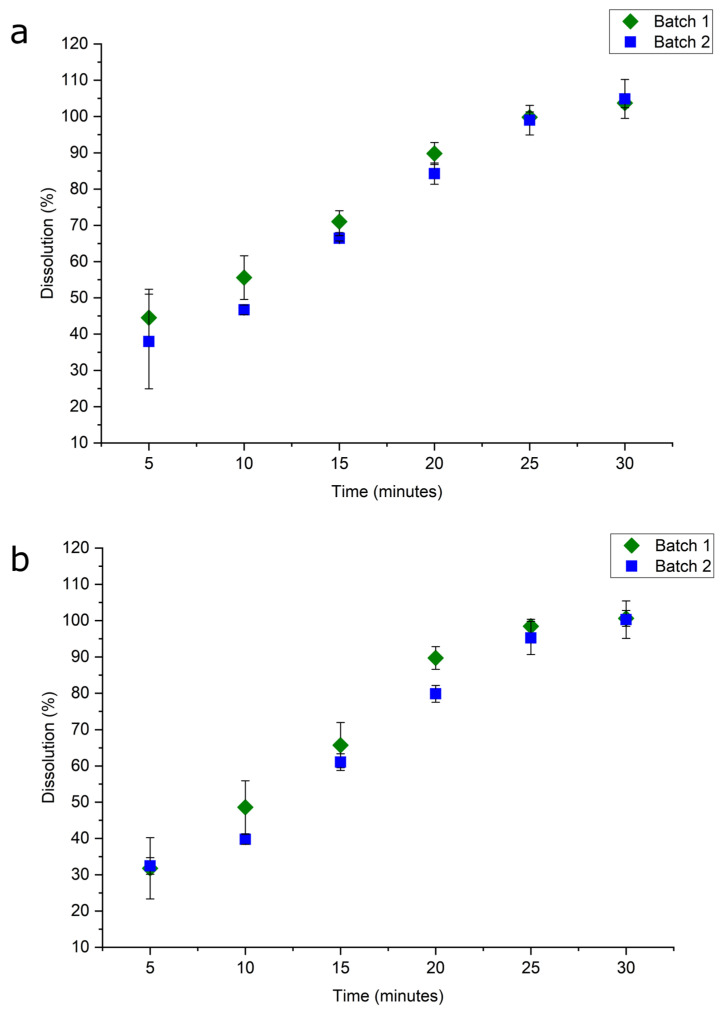
Results of the dissolution test obtained via (**a**) UV-spectroscopy and (**b**) HPLC tests of the API batches.

We then followed the dissolution of nicomorphine in the two batches by using UV spectroscopy and HPLC. The in vitro dissolution profiles (Figure 5) of the granulate including the API (compositions from the producer not described) show that both analytics (UV and HPLC) display the same trend of drug release (nicomorphine). Complete drug release happens within 30 min; however, the final concentration values differ slightly between the two methods (Figure 5). The error bars are larger in the early stage of the dissolution for both methods (Figure 5), i.e., the first 10 min, and afterwards appear more stable for UV. The HPLC display some discrepancies in the dissolution values at the end of the release, i.e., in the minutes range from 25 to 30 min. Thus, the dissolution evaluations for both methods appear to be the same with slight differences at the beginning and the end of the API release. 

However, using the UV method enables a quick analysis of samples that can be performed immediately following the dissolution experiment, and there is no preparation time for the mobile phase solutions. In some cases, transferring the solutions from the test tubes to the HPLC vials can increase the potential for analytical errors. While one might think that analysis of dissolution samples via HPLC would always be the most efficient and effective method, analysis via UV spectroscopy does provide immediate data and trends, as well as significant cost savings. On the other hand, there are situations in which HPLC offers advantages over UV spectroscopy. Depending upon wavelength, the type of dosage form (e.g., capsules), and the dissolution medium being used, the capabilities of UV spectroscopy may be limited. Separation via HPLC might be needed to characterize degradation products and excipients that absorb at the same wavelength as the active one. Evidently, the porosities in both granulates batches are the same; otherwise, differences in the pore diameter would cause differences in the in vitro dissolution profiles. The values cross over 100% drug release in the case of the HPLC indicates some uncertainty with this method relating to the molecular separations.

### 2.5. DSC

**Figure 6:**
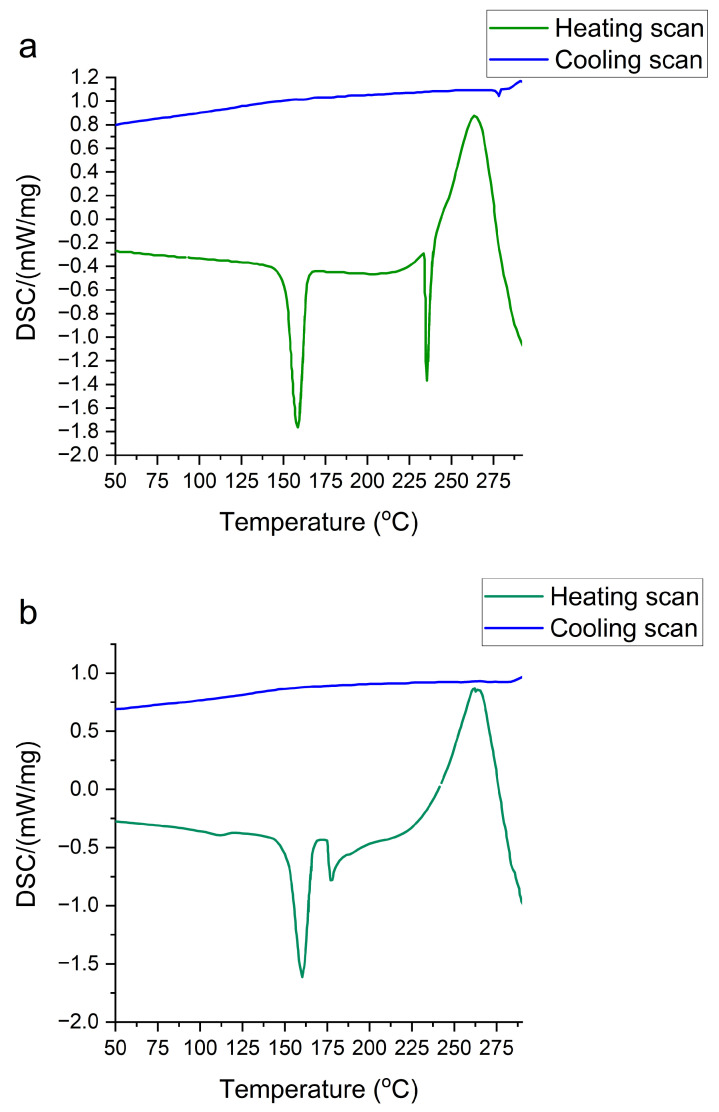
Differential scanning calorimetry (DSC). The thermal behavior during heating and cooling of the primary API (nicomorphine) powders from two different manufactures. (**a**) The first API (batch 1) from one manufacturer and (**b**) the second APIs (batch 2) from another manufacturer.

Finally, the thermotropic behavior of the two batches was observed via DSC. For each batch, one thermogram is shown in Figure 6. The sample of nicomorphine from the first supplier shows a sharp endothermic peak at 158.8 °C during heating (Figure 6a). The onset starts at 151.6 °C and ends at 164.0 °C. The second endothermic peak appears at a temperature of around 235.4 °C. The sharpness of the peaks displays the purity of the samples and reports only one polymorphic structure. In addition, we obtained an exothermic transition at 261.3 °C before melting. The cooling signal remains at a stable state without additional transitions.

The other batch of nicomorphine displays an endothermic sharp peak at 160.3 °C during heating (Figure 6b). The onset starts at 152.5 °C and ends at 166.0 °C. This first transition shows the same behavior as obtained with previous batches (see Figure 6a). However, the second endothermic transition is shifted to a temperature of 177.2 °C. The shift indicates the presence of impurity, since the transition signal does not appear as sharp as in the first batch (Figure 6b). However, any kind of impurities should cause melting point depression. In addition, the main exothermic transition remains the same at 261.3 °C and the cooling signal appears stable without showing any additional transition. In general, the DSC traces show the same trend as the SAXS traces, already concluding the presence of some minor impurity. This could be either with an additional polymorphic change as a polymorphic mixture and the presence of an additional polymorph or a minor chemical impurity, which is obviously not detectable with the standard chemical analytics of Raman and FTIR. 

## 3. Materials and Methods

### 3.1. Materials

The two batches of nicomorphine were obtained in powder form from the G.L. Pharma Company (Lannach, Austria) with the question of purity. The two batches were produced by two different suppliers, and the company wanted to decide on one of them.

### 3.2. Methods

#### 3.2.1. SWAXS

The laboratory’s S3-Microcamera (Hecus X-ray Systems, Graz, Austria) for SWAXS was used to perform the measurements. The SWAXS (SAXS and WAXS) instrument is equipped with a high-brilliance micro-beam system. The operation power is 50 kV and 1 mA (50 W), and the optics is point-focus (FOX3D, Xenocs, Grenoble, France) using a 1D detection system where the data are delivered automatically with the acquisition Hecus 3D View, Easy SWAXS 3.1 software. The detectors used for recording the SWAXS data were two separate one-dimensional detectors (PSD-50, Hecus X-ray Systems, Graz, Austria) having the angular scale for SAXS (0.06° < 2θ < 8°) and for WAXS (17° < 2θ < 27°). An SAXS range calibration was performed by measuring silver behenate (with known lamellar spacing of 5.838 nm), whereas the WAXS range calibration was defined by measuring p-bromo-benzoic-acid. Thus, the scattering angle could be defined to respective detector pixel signals. The symbol λ refers to the X-ray wavelength with a value of 1.54 Å. The q vector is built to serve a wavelength-independent scale, i.e., the x or q-scale. The q-scale is defined by the relation q = 4π sin θ/λ, where θ is the scattering angle. The measurement exposure time was 10 min, and all measurements were conducted at room temperature. 

#### 3.2.2. Attenuated Total Reflectance Fourier Transform Infrared Spectroscopy (ATR-FTIR)

For the infrared measurements, attenuated total reflectance Fourier transform infrared spectroscopy (ATR-FTIR) was performed with a VERTEX 70 (Bruker, Rheinstetten, Germany). The instrument possessed an ATR unit (MVP Pro Star, Diamond crystal Bruker, Rheinstetten, Germany) and a DLaTGS detector. For better statistics, multiple measurements were observed and analyzed with IR spectroscopy. 

#### 3.2.3. Raman

A Raman Station 400 F spectrometer (Perkin Elmer, Waltham, MA, USA) using backscattering geometry was used for recording the Raman spectra. The instrument possessed an echelle spectrograph with a cooled (at −50 °C) CCD detector and a laser source (350 mW near-infrared (785 nm)), delivering a diameter of ca. 100 μm and 100 mW. The triplet measurements were performed with the exposure time of 1 s. Collection of the spectra was performed in a mapping mode. To efficiently scan a maximum sample area, an automatic movement at a defined lateral resolution under the laser irradiation source was performed. All measurements were conducted sufficiently in statistical terms to avoid potential statistical artifacts.

#### 3.2.4. Dissolution

Dissolution analytics were performed using a United States Pharmacopoeia (USP) apparatus I (Pharma Test Type PTWS III C, Pharma Test Apparatebau AG, Hainburg, Germany) at a rotation speed of 100 rpm and a temperature of 37 °C. The quantification of the nicomorphine followed, via reversed-phase high performance chromatography (RP-HPLC) and ultraviolet (UV) spectroscopy. A quantitative filter paper was used for filtration of the collected sample for UV evaluation, and in the case of HPLC evaluation, filtration was performed through 0.45 μm regenerated cellulose membranes.

#### 3.2.5. Differential Scanning Calorimetry (DSC)

The DSC 204 F1 Phoenix instrument (Netzsch, Selb, Germany) was used for the DSC measurements. Aluminum pans were filled with the powder samples (4 to 5 mg) and closed via cold welding and the lid was pierced. A heating rate of 5 °C/min was applied by heating the samples in a temperature range from 20 to 300 °C. At the end of the heating procedure, the samples were kept and equilibrated at 300 °C for 5 min. Afterwards, the samples were cooled down to 20 °C using the same rate of 5 °C/min. A flow of analytical-grade nitrogen (20 mL/min) was applied. Triplet measurements were performed for each batch from each supplier/producer.

## 4. Conclusions

A better understanding of the fundamental physicochemical behavior of pharmaceutical molecules could be key to significantly increasing the success rate of today’s drug structure discovery. Pharmaceuticals on the market consist mainly of molecular crystals in the solid state. The arrangement of molecular crystals determines the physical and chemical properties of pharmaceuticals and thus affects the formulation and processing of pharmaceutical products in solid doses, as well as the stability and speed of dissolution, which are the key properties of a drug. 

In our work, Raman and FT-IR spectra excluded chemical contamination of both nicomorphine samples, whilst X-ray morphological analysis, especially in the SAXS region, could identify additional symmetric peaks as differences between the two samples, reflecting a polymorphic structural change. Additionally, the DSC analysis confirmed the physical differences between the studied samples from two different manufacturers. Since only physical and not chemical differences were found in the samples (identified using SAXS), it can be concluded that some polymorphic reorganization occurred. It is evident in some initial traces, where additional Bragg peaks are identified only in the SAXS region, indicating that the beginning of the transformation of this polymorph first occurs at larger distances of the crystal lattice, visible on the SAXS scale. 

The advantages of the SAXS method in pharmaceutical research and development are discussed in various works on drug delivery and soft materials [25]. However, since an API is the main part of a pharmaceutical product, supporting API studies by providing additional parameters and information makes the method even more attractive. Good API quality leads to a good pharmaceutical product. An API can consist of several different crystalline forms that then form a polymorph through the process of polymorphism. In general, API polymorphism remains a major challenge for the pharmaceutical industry, mainly because it is difficult to follow or predict the nature of polymorphism. Crystallization of the molecule into one or more crystalline forms, or combining with other molecules to form a stable co-crystal, are examples of the nature of polymorphism complexity. However, several polymorphic forms of a particular API could exist for long or short times due to their stability. Thus, different polymorphic forms can have characteristics that differ from each other. Each polymorph can have different chemical, thermal, physical or mechanical properties, which can affect different stability, dissolution, solubility, bioequivalence, bioavailability and production of the API [26]. Therefore, it is necessary to first characterize the polymorphic form of the API to understand its nature. If the shape is not compatible with the formulation, it can cause problems not only in the manufacturing process but also in the applied therapy. Hence, the quality and purity of API polymorphs are the basis for improving the stability and efficiency of pharmaceutical products and also avoiding possible side effects.

However, there are already reports and works about [17,26,27,28,29,30,31,32,33,34,35,36,37,38,39,40,41,42,43] polymorphism in solids using mainly X-ray powder diffraction (XRD), presenting it as a common phenomenon in drugs, which can lead to compromised quality due to changes in their physicochemical properties. Due to these issues, an increase in resources on this topic is crucial, including new and deeper methods, since polymorphism must be controlled to prevent possible ineffective therapy and/or improper dosage. In addition, this also leads us to strengthen and support this area of analytics, since few mandatory tests for the identification and control of polymorphism in medications are currently available, which can result in serious public health concerns. Thus, more commitment is necessary by regulatory and quality control authorities to monitor polymorphism for all commercial drugs and not only for research and development [44,45,46,47,48,49,50,51,52,53,54,55]. The stability of drugs is always an important issue and aging or instability are for many products still not sufficiently investigated at the nanoscale, where changes in polymorphism with time can occur without their timely identification [26]. To improve the monitoring of a polymorphism, new techniques should be welcomed to support and improve analytics, since monitoring includes the control of polymorphism in raw materials, manufacturing steps and finished products by the end of the shelf life of the drug. In this manner, possible public health concerns linked to polymorphism in medicines can be avoided.

Based on many years of experience, we have come to the conclusion that polymorphic changes and restructuring are much more often encountered and identified in applied research, i.e., industry, than they are published. The reason for this is that industry research is often confidential, whereby newly discovered polymorphs are not published and do not enter the literature base, which in our opinion should be changed for the general scientific benefit. We also waited for a 5-year confidentiality period to pass before we could publish this paper, but we are happy to share this information with the scientific world. According to our analyses, the company G.L. Pharma could leave the supplier/producer with new polymorphic API changes due to instability. We know that polymorphic changes can be important for the stability, release, aging, bioavailability, etc., of pharmaceutical products; with API physico-structural differences, a drug may effect lower bioavailability in the human body and thus cause more side effects.

Therefore, the message of this paper is simply to show that the use of additional non-standard methods with extended resolution scale for structure quality (such as SAXS) can be very useful for detecting such objections in the drug and thereby avoiding possible side effects of the drug in human body. The fact is that SAXS is also used like WAXS, XRD or other methods [17,27,28,29,30,31,32,33,34,35,36,37,38,39,40,41,42,43,44,45,46,47,48,49,50,51,52,53,54,55,56] in the study of polymorphism [57,58,59,60,61,62,63,64,65,66,67,68,69,70,71,72,73,74,75,76,77,78,79,80,81,82,83,84,85,86], but these are mainly self-assembling systems, so it has not yet become a standard method and part of the GMP in pharmaceutical industry. Therefore, we believe that the introduction of new methods into the standard pharmacy procedure should be considered for better analysis and quality control of medicines.

## Data Availability

The data presented in this study are available on request from the corresponding authors.
